# Traveling Wave Enantioselective Electron Paramagnetic Resonance

**DOI:** 10.1021/acs.jpclett.3c00519

**Published:** 2023-05-09

**Authors:** Manuel Donaire, Nicolas Bruyant, Geert L. J. A. Rikken

**Affiliations:** † Departamento de Física Teórica, Atómica y Óptica and IMUVA, Universidad de Valladolid, Paseo Belén 7, 47011 Valladolid, Spain; ‡ Laboratoire National des Champs Magnétiques Intenses UPR3228 CNRS/EMFL/INSA/UGA/UPS, 31400 Toulouse and 38042 Grenoble, France

## Abstract

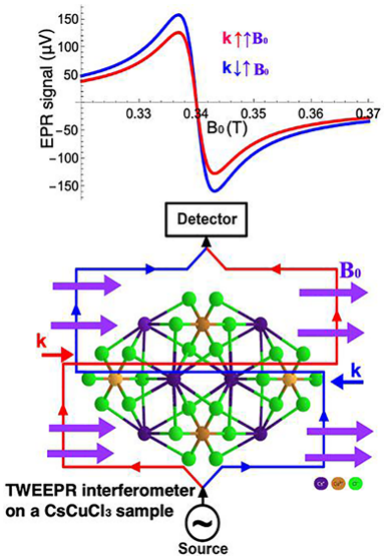

We propose a novel method for enantioselective electron paramagnetic resonance (EPR) spectroscopy based on magneto-chiral anisotropy. We elaborate a theoretical model to estimate the strength of this effect and propose a dedicated interferometer setup for its experimental observation.

Electron paramagnetic resonance (EPR) spectroscopy is a powerful technique to study the local environment and the dynamics of spin-carrying entities, like transition metal ion complexes and organic radicals.^1^ Also, those systems that do not intrinsically carry a spin can still be studied by EPR through spin-labeling, i.e., by selectively adding-on a spin carrying probe.^2^ Many of the systems studied by EPR are chiral; i.e., they exist in two nonsuperimposable forms (enantiomers) that are each other’s mirror image, particularly in biochemistry where enzymes, metalloproteins, membranes, etc., are chiral subjects of intense EPR activity.^3^ However, EPR is universally believed to be blind to chirality. Here, we present the paradigm shift that EPR in the proper configuration is intrinsically sensitive to chirality because of magneto-chiral anisotropy (MChA).

MChA corresponds to an entire class of effects in chiral media under an external magnetic field, which show an enantioselective difference in the propagation of any unpolarized flux that propagates parallel or antiparallel to the magnetic field. This difference has its origin in the simultaneous breaking of parity and time-reversal symmetries as a result of the chirality of the media and the magnetization induced by the external magnetic field, respectively. Generally, such a difference manifests itself in the velocity or the attenuation of the flux. MChA has been predicted since 1962 in the optical properties of chiral systems in magnetic fields^4−8^ and was finally observed in the 1990s.^9−11^ Nowadays, it is observed across the entire electromagnetic spectrum, from microwaves^12,13^ to X-rays.^14,15^ The existence of MChA was further generalized to electrical transport^16^ (in carbon nanotubes,^17^ organic conductors,^18^ metals,^19−21^ and semiconductors^22^), to sound propagation,^23^ and to dielectric properties.^24^

EPR is basically a strongly resonant form of magnetic circular dichroism and magnetic circular birefringence,^25^ effects well-known in the optical wavelength range, where they however only represent small perturbations of the optical properties of the medium. By analogy, one should expect that MChA can manifest itself also in EPR of chiral media. This expectation can be formalized by the observation that the EPR transition probability *P* induced by a propagating electromagnetic field between the spin levels of a chiral medium in a magnetic field is allowed by parity and time-reversal symmetry to have the form
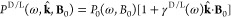
1In this equation, **B**_0_ is an external and constant magnetic field, *P*_0_ is the leading order transition probability between the Zeeman levels, common to both enantiomers, the handedness of the medium is represented by D (right) and L (left) with γ^D^ = −γ^L^, and **k̂** is a unitary vector in the direction of the wave vector of the electromagnetic field driving the transition whose frequency ω is of the order of μ_B_*B*_0_/ℏ. This shows that the EPR transition probability is enantioselectively modified when probed by an electromagnetic wave traveling parallel or antiparallel to the magnetic field, an effect that we shall call traveling wave enantioselective EPR (TWEEPR). TWEEPR is quantified by the anisotropy factor *g*_T_^D/L^, which represents the relative difference between the transition probabilities of both enantiomers,
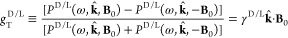
2As spin is related to the absence of time-reversal symmetry and chirality is related to the absence of parity symmetry, one might expect that the two are decoupled and that *g*_T_^D/L^ is vanishingly small, thereby reducing TWEEPR to an academic curiosity. However, below, we will show through a model calculation that, because of the ubiquitous spin–orbit coupling, TWEEPR represents a significant and measurable fraction of the EPR transition probability for realistic chiral systems and that its anisotropy factor is not much smaller than that of optical MChA. Lastly, we will describe a dedicated TWEEPR setup.

## Theoretical Model

As for the spin system of our model calculation of TWEEPR, without loss of generality, we have chosen a crystalline quasi-octahedral Cu(II) chiral complex because this ion is one of the most extensively studied systems by EPR, it has the largest spin–orbit coupling among the first row transition metals, and it has the simplest energy diagram. Its electromagnetic response is attributed to a single unpaired electron that, in the 3d^9^ configuration of the Cu(II) complex, behaves as a hole of positive charge + *e*. We model the binding potential of the hole by that of an isotropic harmonic oscillator that represents the rest of the ion and is perturbed by the chiral potential *V*_C_^D/L^ that results from its interaction with the chiral environment of the crystal lattice and by the spin–orbit coupling. In turn, as we will show, this model allows us to find analytic expressions for both the optical and the EPR magnetochiral anisotropy parameters, *g*_O_^D/L^ and *g*_T_^D/L^, respectively, in terms of the parameters of the model, both being proportional to the chiral coupling. Our model can thus relate *g*_T_^D/L^ to its optical analogue *g*_O_^D/L^. The latter is experimentally determined for several systems. In particular, for CsCuCl_3_, both MChD^26^ and EPR^27^ have been reported, which makes it currently the only Cu(II) system for which our model can give a quantitative prediction. This approach thereby results in a generic analytical expression for *g*_T_^D/L^ in terms of the parameters of our model and in a semiempirical and quantitative prediction for *g*_T_^D/L^ for this particular material in terms of its experimental optical MChD. The latter can be extended to any material for which optical MChD has been determined. Nonetheless, in the calculations, we constrain ourselves to the single-molecule approximation, neglecting this way spin–spin and near field interactions between nearby molecules in the crystal. Below, we detail our model, which is a variant of Condon’s model for optical activity,^28,29^ and its extension to optical magnetochiral birefringence.^30^

The Hamiltonian describing the system is given by *H* = *H*_0_ + *V*_C_^D/L^ + *V*_SO_, with
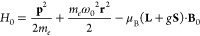
3
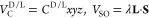
4where **r**, equal to (*x*, *y*, *z*) in Cartesian coordinates, and **p** are the position and kinetic momentum vectors of the harmonic oscillator, ω_0_ is its natural frequency, **L** and **S** are their orbital and spin angular momentum operators, respectively, *C*^D^ = −*C*^L^ is the right/left-handed chiral coupling, *g* ≃ 2 is the Landé factor, λ (≃−0.1 eV) is the spin–orbit (SO) coupling parameter, and **B**_0_ ≡ *B*_0_**ẑ** is the external magnetic field. The interaction with an electromagnetic plane-wave of frequency ω, propagating along **B**_0_, is given in a multipole expansion by
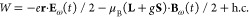
5where **E**_ω_(*t*) = *i*ω**A**_ω_*e*^–*i*ω*t*^ and **B**_ω_(*t*) = *in̅***k** ∧ **A**_ω_*e*^–*i*ω*t*^ are the complex-valued electric and magnetic fields in terms of the electromagnetic vector potential, **A**_ω_, evaluated at the center of mass of the ion. Note that the field incident on a molecule of the complex is the effective field which propagates throughout the medium with an effective index of refraction *n̅*. Hence, it is the effective wavevector *n̅***k** that appears.

**Figure 1:**
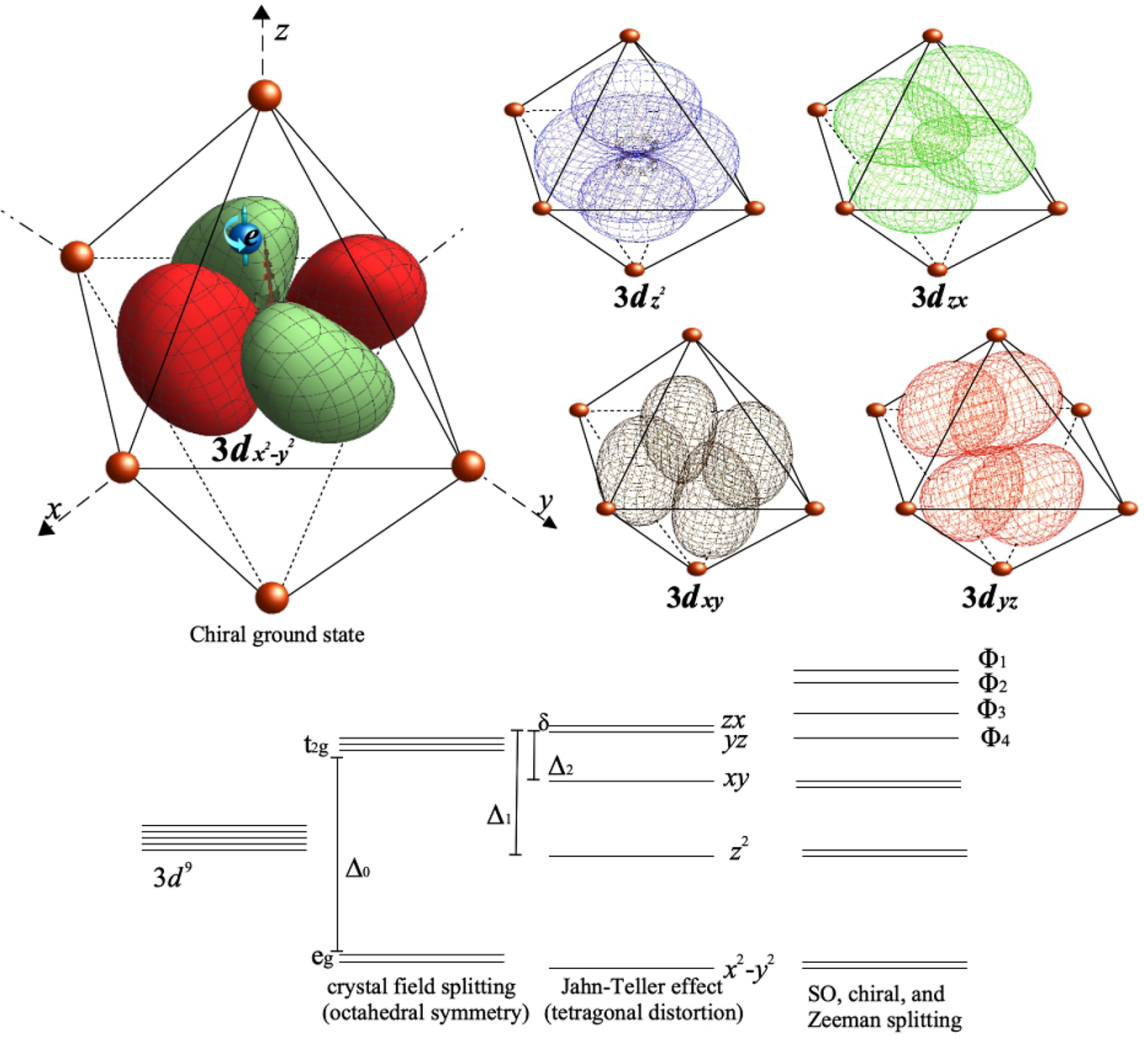
Energy levels of Cu(II) in a chiral quasi-octahedral configuration. Approximate experimental values are Δ_0_ ≃ 1.5 eV, Δ_1_ ≃ 0.5 eV, and Δ_2_ ≃ 0.23 eV.

In our model, the 3d orbitals are represented by linear combinations of the *n* = 2, *l* = 2 states of the isotropic harmonic oscillator; see the Supporting Information. Essential to the original Condon model was the anisotropy of the harmonic oscillator, which removes all axis and planes of symmetry. In our model, such an anisotropy is provided by the interaction of the ion with the surrounding ligands of the complex, which in the case of CsCuCl_3_ form a quasi-octahedral structure. In the first place, that interaction causes the elongation of the 3d orbitals which lie along the *z*-axis, opening an optical gap Δ_0_. Also, in conjunction with the Jahn–Teller distortion and the helical configuration of the Cu(II) ions, it removes the degeneracy between the orbitals lying on the *xy* plane and generates a small energy gap δ between the states *d*_*zx*_ and *d*_*yz*_ with λ ≫ δ. The ground state of the Cu(II) ion in the octahedral configuration Ψ is, at finite temperature and subject to a magnetic field, a linear combination of the doublet *d*_*x*^2^–*y*^2^_ ⊗ {↑, ↓},
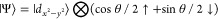
6where θ, being a function of *B*_0_ and the temperature, is the angle between the magnetization of the sample and **B**_0_. For EPR, spin-flip takes place at a resonance frequency Ω = *g*μ_B_*B*_0_/ℏ when the up ↑ component of Ψ turns into |Φ⟩ = |*d*_*x*^2^–*y*^2^_⟩ ⊗ ↓, with probability proportional to cos^2^θ/2, and the down ↓ component turns into |Φ′⟩ = |*d*_*x*^2^–*y*^2^_⟩ ⊗ ↑ with probability proportional to sin^2^θ/2. The net absorption probability is thus proportional to cos^2^θ/2 – sin^2^θ/2 = cos θ and hence to the degree of magnetization along **B**_0_. At *B*_0_ = 1 T, Ω corresponds to an energy 150 μeV. In contrast, optical absorption happens at an energy Δ_0_ ≃ 1.5 eV toward the quadruplet {*d*_*zx*_, *d*_*yz*_} ⊗ {↑, ↓}. Applying standard perturbation theory with the spin–orbit and the Zeeman potentials upon this quasidegenerate quadruplet, we end up with the four states ϕ_*i*_, *i* = 1, ..., 4, as appear in the energy diagram represented in Figure 1; a brief description can be found in the Supporting Information. It is of note that these states play a crucial role in the E1M1 transitions of both EPR and its optical analogue.

## Theoretical Results

Using up to fourth order time-dependent perturbation theory on *V*_SO_, *V*_C_, and *W*, in the adiabatic regime, our model allows us to calculate the standard EPR and optical transition probabilities, as well as the MChA corrections to both of them, with the latter two being both proportional to *C*^D/L^. As for *g*_T_^D/L^, the probability difference in the denominator of eq 2 is an enantioselective E1M1 transition, whereas the denominator equals in good approximation the leading order M1M1 transition, *g*_T_^D/L^ = *P*_E1M1_^D/L^/*P*_M1M1_|_ω_ ≈ Ω, with
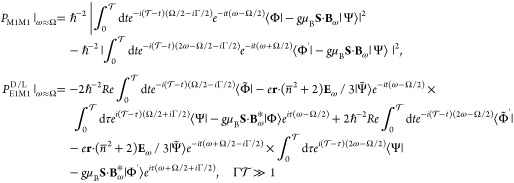
7where Γ is the line width of EPR absorption,  implies continous radiation, and the states Ψ̃, Φ̃, and Φ̃′ are dressed with the states ϕ_*i*_, *i* = 1, .., 4, on account of the spin–orbit and chiral interactions. Using a linearly polarized microwave probe field in eq 7, the resultant expression for the TWEEPR anisotropy factor reads
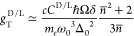
8where the second factor on the right-hand side describes the effect of the refractive index on the local electric field and the wavevector. It is worth noting that the aforementioned dependence on magnetization, ∼cos θ, cancels out in the ratio between probabilities. For further details, see the Supporting Information.

The values for the unknown parameters in eq 8 can be deduced comparing the predictions of the model with the experimental results for optical MChD^26^ and EPR^27^ in CsCuCl_3_. In particular, we can estimate *g*_T_^D/L^ from the data on the nonreciprocal absorption coefficient in optical MChD, 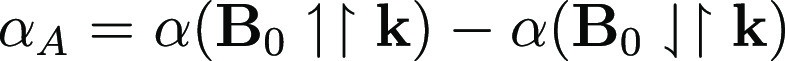. The calculation is as follows. In terms of the E1M1 absorption probability at resonance, ω = Δ_0_/ℏ, α_A_ reads
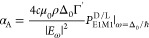
9where Γ′ is the line width of optical absorption and ρ is the molecular number density of the complex. Using our model, a calculation analogous to that for *P*_E1M1_^D/L,EPR^, but for its optical counterpart, *P*_E1M1_^D/L,O^ (see the Supporting Information), allows as to express *g*_T_^D/L^ in eq 8 in terms of α_A_,
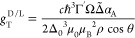
10where Δ̃^–1^ = Δ_0_^–1^ + Δ_2_^–1^ – 3Δ_1_^–1^ is the inverse of an effective energy interval which takes account of the optical transitions to intermediate states; see Figure 1. It is of note that, whereas the magnetic transition is driven in EPR by the spin operator [eq 7], it is driven by the orbital angular momentum in the optical case. In turn, this causes MChD to be stronger in the optical case and proportional to the degree of magnetization cos θ, which can be approximated by cos θ ≈ μ_0_*B*_0_/*k*_B_*T*.^32,33^ The optical MChA parameter, *g*_0_^D/L^, has an analogous expression to that in eq 2 with ℏω ≈ Δ_0_ being proportional to α_A_. Hence, our model allows us to estimate its upper bound, *g*_0_^D/L^ ≤ (*cC*^D/L^δ cos θ)/(*m*_*e*_ω_0_^3^Δ̃) (see the Supporting Information), from which *g*_T_^D/L^/*g*_0_^D/L^ ≳ (ℏΩΔ̃)/(Δ_0_^2^ cos θ). Note that, since both Ω and cos θ are proportional to *B*_0_, the ratio between EPR and optical MChA factors is independent of the field strength.

Finally, substituting the experimental values for CsCuCl_3_ of all the variables in eq 10, for *B*_0_ = 14 T at a temperature of 4.2 K, we obtain *g*_T_^D/L^ ≈ 1.5·10^–2^, which is small but not beyond the resolution of high field EPR spectrometers. For an X band EPR spectrometer (*B*_0_ = 0,35 T), this means *g*_T_^D/L^ ≈ 3·10^–4^, which will require a different approach, as we discuss below.

## Experimental Implementation

**Figure 2:**
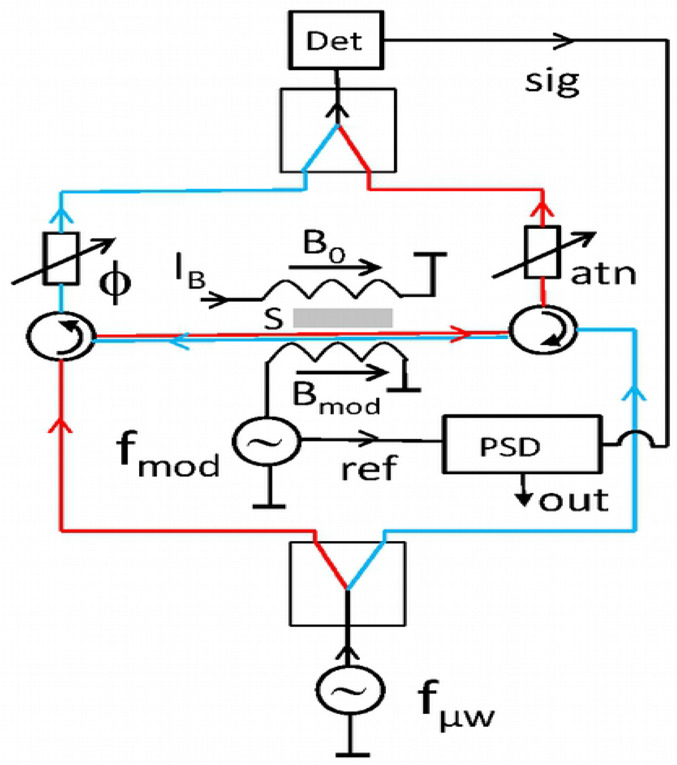
Schematic setup of the TWEEPR interferometer. The waves counterpropagating through the sample S are depicted in red and blue.

In commercial EPR spectrometers, resonant standing wave cavities are used to enhance sensitivity. Such a cavity can be regarded as containing equal amounts of traveling waves with **k** and −**k**. The MChA γ^D/L^ term in eq 1 can therefore not give a net contribution to the resonance in such a configuration. For this term to be observed, a traveling wave configuration should be used. Such configurations are not unknown in EPR; several reported home-built EPR spectrometers have used one-pass transmission configurations.^34−39^ Sensitivity for such a traveling wave configuration can be enhanced by means of a Mach–Zehnder interferometer^40^ or a unidirectional ring resonator.^41,42^ In such a configuration, MChA can be obtained as the difference between the microwave transmissions for the two opposing magnetic field directions, 
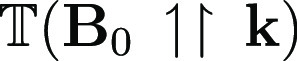
 and 
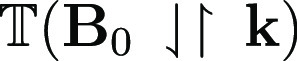
, similar to what was realized in the optical case.^11^ As the EPR lines can be quite narrow, the two oppositely oriented magnetic fields should have the same magnitude with high precision, which requires a tight control of this field, possibly with another EPR or NMR feedback circuit. Stabilizing a field this way can be quite time-consuming, and TWEEPR, being a small difference on the already small EPR absorption, the extensive signal-averaging through field alternations that would be required to obtain a good signal-to-noise-ratio makes such an approach impractical. We therefore propose another approach in the form of an X band microwave interferometer that removes the normal EPR contribution from the output signal, through destructive interference between counterpropagating waves through the sample at a fixed magnetic field, as illustrated in Figure 2. This leaves ideally only the TWEEPR contribution. By applying an additional small modulation field and using phase sensitive detection (PSD), sufficient sensitivity is obtained to resolve this small contribution. When tuned to total destructive interference at zero field, the interferometer output as given by the PSD is proportional to the TWEEPR response 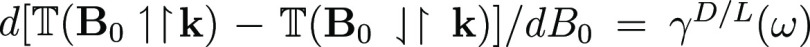. The sensitivity of the interferometer can be further improved by inserting the sample in a unidirectional resonant ring resonator. *Q* factors above 10^3^ have been reported for such configurations^43^ and would bring a corresponding increase in sensitivity. It seems therefore quite feasible that TWEEPR can evolve into a standard characterization technique in the form of standalone dedicated TWEEPR spectrometers. An alternative to this configuration could be the microwave equivalent of the first observation of optical MChA in luminescence,^9^ using pulsed EPR echo techniques^1^ with a similar interferometer setup.

## Discussion and Conclusions

In general, the nonlocal response of a chiral system of size *a* to an electromagnetic wave with wave vector *k* is of the order *ka*, so one could have expected *g*_T_^D/L^/*g*_O_^D/L^ to be of the order ℏΩ/Δ_0_, the relevant spatial length scale for both TWEEPR and optical MChD being the orbital size. This ratio is of the order of 10^–4^, which would have put TWEEPR beyond experimental reach. However, in contrast to the optical absorption, which to zeroth order is independent of the magnetic field, the normal EPR absorption scales with the magnetization of the spin system. Since the MChA corrections are proportional to the magnetization in both EPR and the optical case, the cancellation of the factor cos θ ≪ 1 applies to *g*_T_^D/L^ only, and it appears thereby in the denominator of *g*_T_^D/L^/*g*_O_^D/L^, resulting in eq 10. For room temperature X-band EPR of Cu(II), this results in *g*_T_^D/L^/*g*_O_^D/L^ of the order of 10^–1^, which makes TWEEPR experimentally feasible under those conditions. As a consequence, and in contrast to many other magnetic resonance techniques, going to low temperatures is not necessarily favorable for TWEEPR. Going to higher magnetic field does not affect *g*_T_^D/L^/*g*_O_^D/L^, the increase in Ω being compensated by the concomitant increase of cos θ because of the higher resonance field.

The main results of our model are an analytic expression for the TWEEPR anisotropy factor [eq 8] and an expression for its relationship with the optical anisotropy absorption coefficient [eq 10]. The expression in eq 8 shows that *g*_T_^D/L^ has a linear dependence on the magnetic field strength (through Ω) and on the chirality (through *C*^D/L^), as predicted by symmetry arguments. The dependence on the spin–orbit coupling does not appear explicitly, because we have considered the case for Cu(II), where the level splitting δ is much smaller than the SO coupling λ. In the inverse case, *g*_T_^D/L^ would be proportional to λ instead. Adapting the calculation to other chiral transition metal complexes is conceptually straightforward and should result in an expression similar to eq 8, apart from numerical factors of order unity. A rather different case is represented by chiral organic radicals, where the unpaired electron is delocalized on one or more interatomic bonds and a different microscopic model should be used for the calculation of *g*_T_^D/L^. One might however expect that such differences apply also to the calculation of *g*_O_^D/L^ for such radicals, preserving a relationship similar to that in eq 10.

## Supporting Information Available

Theoretical model used for the calculations; explicit expressions for the transition probabilities that enter the anisotropy factors in EPR and optical MChD; explanation of the limitations of our model (PDF)
