# Supplementary material for: Traveling Wave Enantioselective Electron Paramagnetic Resonance
Source: J Phys Chem Lett. 2023 May 9;14(19):4504–9. doi: 10.1021/acs.jpclett.3c00519 (PMC10201572; doi:10.1021/acs.jpclett.3c00519)
Supplement: Supplementary file 1 — jz3c00519_si_001.pdf [file jz3c00519_si_001.pdf]

Supporting Information for Traveling Wave Enantio-selective Electron Paramagnetic Resonance

M. Donaire[1*], N. Bruyant[2] and G.L.J.A. Rikken[2]

[1]*Departamento de Física Teórica,*
Atómica y Óptica and IMUVA,
Universidad de Valladolid,
Paseo Belén 7, 47011 Valladolid, Spain
[2]*Laboratoire National des Champs Magnétiques Intenses*
UPR3228 CNRS/EMFL/INSA/UGA/UPS,
31400 Toulouse & 38042 Grenoble, France
[*]Email: manuel.donaire@uva.es

Outline

In this supporting material we describe the theoretical model used in our calculations, Note 1; we provide explicit expressions for the transition probabilities that enter the anisotropy factors in EPR, Note 2a, and optical MChD, Note 2b, and we elaborate on the estimate of the relationship between both factors, Note 2c; we finalise with some comments on the limitations of our model, Note 3.

Note 1. Fundamentals of the model

As outlined in the Letter, in order to estimate the MChA factors of a chiral Cu(II) complex, we consider a variant of the one-electron model proposed by Condon for the study of natural optical activity in chiral compounds [1, 2]. The total Hamiltonian of our model is $H = H_0 + V_C^{D/L} + V_{SO}$, where $H_0 = \frac{p^2}{2m_e} + \frac{m_e\omega_0^2 r^2}{2} + V_Z$ is the unperturbed Hamiltonian, with $V_Z = -\mu_B(\mathbf{L}+g\mathbf{S})\cdot\mathbf{B}_0$ being the Zeeman potential; and $V_C^{D/L} = C^{D/L}xyz$, $V_{SO} = \lambda\mathbf{L}\cdot\mathbf{S}$ being the chiral potential and the spin-orbit coupling, respectively. We stick to the nomenclature used in the Letter. The chiral Hamiltonian, $V_C^{D/L}$, results from the electrostatic interaction of the ion with the chiral configuration of the ligands in the complex, and produces the necessary parity asymmetry which is at the origin of natural optical activity. The orbital contribution of the Zeeman potential was added in Ref.[3] to the original Condon's model to estimate the magneto-chiral birefringence of diamagnetic chiral compounds. In order to account for magnetochiral dichroism (MChD) in a paramagnetic complex, we introduce here the spin contribution to the Zeeman potential as well as the spin-orbit coupling. In contrast to the approach in Ref.[3] and for simplicity, we consider an isotropic harmonic oscillator, whereas the anisotropy caused by the crystal field is introduced in an effective manner through the energy intervals between the $3d$ orbitals, as depicted in Fig.1 in the Letter.

The eigenstates of H_0 are labeled with the eigenvalues of the orbital angular momentum and spin operators, $\{|n_L, n_R, n_z\rangle\} \otimes \{\uparrow, \downarrow\}$ [6], upon which $V_C^{D/L}$ and V_{SO} act perturbatively. In a Cu(II) complex, the chromophoric charge is the unpaired electron of the $3d^9$ electronic configuration which behaves as a hole of positive charge. In the absence of ligands, the $3d$ orbitals of the ion can be represented approximately by the $n = 2$, $l = 2$ states of the harmonic oscillator of our model. However, the ligands' fields affect the electronic configuration of the ion, removing the degeneracy of the d-states. In particular, for octahedral

coordination geometries around the ion, the set of d-orbitals splits into doubly degenerate e_g orbitals, $d_{x^2-y^2}$ and d_{z^2}, and triply degenerate t_{2g} orbitals, d_{xy}, d_{yz} and d_{zx}. The energy interval between e_g and t_{2g} states, Δ_0, lies in the visible region of the spectrum, $\Delta_0 \simeq 1.5$ eV. As a result, the e_g orbitals become the ground states, and can be approximated by linear combinations of $l = 2$, $m_l = 0, \pm 2$ eigenstates of the harmonic oscillator. The fact that the chromophoric charge in the e_g states cannot rotate into any other orbital leads to an effective quenching of the orbital angular momentum of the ground state. Below a certain temperature, an additional Jahn-Teller (JT) distortion takes place when the ligands along one of the axes, say the z-axis, move away from the ion in order to minimize the electronic repulsion, giving rise to the complete removal of the degeneracy in the e_g level, and to a partial lifting of the degeneracy in the t_{2g} orbitals. The isotropy of the system is thus broken and the ground state becomes unique, up to spin degeneracy. For the particular case of the $CsCuCl_3$ crystal, the bonds along the z-axis get elongated and the ground state is the $d_{x^2-y^2}$ orbital. Fig.1 in the Letter depicts the energy splitting of the distorted d-orbitals, including the approximate values of the energy intervals. Lastly, the JT distortion in conjuntion with the helical deformation of the crystal along the c-axis, of coordiates [1,1,1] in the local axis basis, removes the degeneracy between the orbitals lying on the xy plane in a small ammount δ. Below, we write the approximate expression of the $3d$ orbitals in terms of the harmonic oscillator eigenstates, $\{|n_L, n_R, n_z\rangle\}$, together with their corresponding energies,

$$\begin{aligned}
|d_{zx}\rangle &= (|0,1,1\rangle - |1,0,1\rangle)/\sqrt{2}, \quad \mathcal{E} = \Delta_0, \\
|d_{yz}\rangle &= i(|0,1,1\rangle + |1,0,1\rangle)/\sqrt{2}, \quad \mathcal{E} = \Delta_0 - \delta, \\
|d_{xy}\rangle &= i(|0,2,0\rangle - |2,0,0\rangle)/\sqrt{2}, \quad \mathcal{E} = \Delta_0 - \Delta_2, \\
|d_{z^2}\rangle &= (|1,1,0\rangle - \sqrt{2}|0,0,2\rangle)/\sqrt{3}, \quad \mathcal{E} = \Delta_0 - \Delta_1, \\
|d_{x^2-y^2}\rangle &= (|0,2,0\rangle + |2,0,0\rangle)/\sqrt{2}, \quad \mathcal{E} = 0.
\end{aligned} \tag{1}$$

Altogether, the crystal field combined with the JT distortion and the helical deformation turns the crystalline structure into a chiral one. In accord with Condon's model, the potential $V_C^{D/L}$ reproduces the electrostatic interaction of the chromophoric charge with the surrounding chiral structure, removing all axes and planes of symmetry from the system. It is through the chiral potential that E1 transitions between the $3d$ orbitals take place in our model. In addition to the above interactions, MChD in EPR requires necessarily the coupling between the spin and the orbital angular momentum of the unpaired electron hole

through the potential V_{SO}, where the coupling constant is $\lambda \approx -0.1$ eV. In particular, the SO interaction together with the Zeeman potential break the quasi-degeneracy between the four states $\{|d_{zx}\rangle, |d_{yz}\rangle\} \otimes \{\uparrow, \downarrow\}$. The diagonalization of $V_{SO} + V_Z$ within the subspace $\{|d_{zx}\rangle, |d_{yz}\rangle\} \otimes \{\uparrow, \downarrow\}$ yields the following eigenstates and eigenenergies for $\lambda \gg \delta$,

$$\begin{aligned}
|\Phi_1\rangle &\approx |1,0,1\rangle \otimes \downarrow + \frac{\delta}{2\lambda}|0,1,1\rangle \otimes \downarrow, \\
\mathcal{E} &\simeq \Delta_0 - \lambda/2 + \hbar\Omega, \\
|\Phi_2\rangle &\approx |0,1,1\rangle \otimes \uparrow + \frac{\delta}{2\lambda}|1,0,1\rangle \otimes \uparrow, \\
\mathcal{E} &\simeq \Delta_0 - \lambda/2 - \hbar\Omega, \\
|\Phi_3\rangle &\approx |0,1,1\rangle \otimes \downarrow - \frac{\delta}{2\lambda}|1,0,1\rangle \otimes \downarrow, \\
\mathcal{E} &\simeq \Delta_0 + \lambda/2 + \hbar\Omega + \frac{\delta^2}{4\lambda^2}(\lambda + \hbar\Omega), \\
|\Phi_4\rangle &\approx |1,0,1\rangle \otimes \uparrow - \frac{\delta}{2\lambda}|0,1,1\rangle \otimes \uparrow, \\
\mathcal{E} &\simeq \Delta_0 + \lambda/2 - \hbar\Omega + \frac{\delta^2}{4\lambda^2}(\lambda - \hbar\Omega).
\end{aligned} \tag{2}$$

In turn, the subspace spanned by $\{\Phi_1, \Phi_2, \Phi_3, \Phi_4\}$ is the set of intermediate states of the transition processes in EPR mediated by the interaction of the spin with the chiral structure of the surrounding charges.

In the following, we apply to our system time-dependent quantum perturbation techniques to compute first the MChA factor in EPR, $g_T^{D/L}$. Next, in order to estimate the value of the unknowns of our model, we compute the anisotropy factor in optical MChD for the same system. Finally, making use of the experimental values available for $CsCuCl_3$ in the literature [4, 5], we estimate the strength of TWEEPR.

Note 2a. MChD in EPR

Let us consider a $CsCuCl_3$ complex, initially prepared in its ground state, and partially polarized along a uniform magnetic field $\mathbf{B} = B_0\hat{\mathbf{z}}$ directed along the z-axis,

$$|\Psi\rangle = |d_{x^2-y^2}\rangle \otimes (\cos\theta/2 \uparrow + \sin\theta/2 \downarrow) \approx \frac{1}{\sqrt{2}}(|0,2,0\rangle + |2,0,0\rangle) \otimes (\cos\theta/2 \uparrow + \sin\theta/2 \downarrow), \tag{3}$$

where we have approximated the actual ground state with the corresponding state of our harmonic oscillator model in the basis $\{|n_L, n_R, n_z\rangle\} \otimes \{\uparrow, \downarrow\}$, and θ is the angle between the magnetic moment of the complex and the z-axis, $\cos\theta = \langle\Psi|2\mathbf{S}|\Psi\rangle \cdot \hat{\mathbf{z}}$. At temperature

T, $\cos\theta \approx \mu_0 B_0/k_B T$ [8]. Under the action of an incident electromagnetic field of frequency ω close to the transition frequency, $\Omega = g\mu_B B_0/\hbar$, and wave vector $\mathbf{k}$ parallel to $\mathbf{B}_0$, the complex gets partially excited towards the state

$$|\Phi\rangle = |d_{x^2-y^2}\rangle \otimes \downarrow \approx \frac{1}{\sqrt{2}}(|0,2,0\rangle + |2,0,0\rangle) \otimes \downarrow, \tag{4}$$

with probability proportional to $\cos^2\theta/2$; and partially de-excited (through stimulated emission) towards the state

$$|\Phi'\rangle = |d_{x^2-y^2}\rangle \otimes \uparrow \approx \frac{1}{\sqrt{2}}(|0,2,0\rangle + |2,0,0\rangle) \otimes \uparrow, \tag{5}$$

with probability proportional to $\sin^2\theta/2$. Since the rest of probability factors are equivalent, the net absorption probability in EPR is proportional to $\cos^2\theta/2 - \sin^2\theta/2 = \cos\theta$, and thus proportional to the magnetization of the complex.

As mentioned in the Letter, from symmetry considerations and in leading order, the numerator and the denominator in the ratio $g_T^{D/L} = \frac{[P^{D/L}(\omega,\hat{\mathbf{k}},\mathbf{B}_0) - P^{D/L}(\omega,\hat{\mathbf{k}},-\mathbf{B}_0)]}{[P^{D/L}(\omega,\hat{\mathbf{k}},\mathbf{B}_0) + P^{D/L}(\omega,\hat{\mathbf{k}},-\mathbf{B}_0)]}$ for $\omega \approx \Omega$ are dominated, respectively, by the electric-magnetic dipole (E1M1) and the magnetic-magnetic dipole (M1M1) transition probabilities, the magnetic transition being driven by the spin operator only. That leads to the approximate expression,

$$g_T^{D/L} \simeq \frac{P_{E1M1}^{D/L}(\omega,\hat{\mathbf{k}},\mathbf{B}_0)}{P_{M1M1}(\omega,\hat{\mathbf{k}},\mathbf{B}_0)}\Big|_{\omega\approx\Omega}. \tag{6}$$

In what follows, we compute the transition probabilities P_{M1M1} and $P_{E1M1}^{D/L}$ for $\omega \approx \Omega$ using time-dependent perturbation theory in the adiabatic regime. This regime is the suitable one for a probe field whose duration is much longer than the typical lifetime for excitation or de-excitation. As in the Letter, the Hamiltonian of the interaction of our system with the microwave probe field reads, in the electric and magnetic dipole approximation, $W = -e\mathbf{r} \cdot \mathbf{E}_\omega(t)/2 - \mu_B(\mathbf{L} + 2\mathbf{S}) \cdot \mathbf{B}_\omega(t)/2 + \text{h.c.}$. In this equation, $\mathbf{E}_\omega(t) = \mathbf{E}_\omega e^{-i\omega t} = i\omega\mathbf{A}_\omega e^{-i\omega t}$, $\mathbf{B}_\omega(t) = \mathbf{B}_\omega e^{-i\omega t} = i\bar{n}\mathbf{k} \wedge \mathbf{A}_\omega e^{-i\omega t}$, are the complex-valued electric and magnetic fields, respectively, with $\mathbf{A}_\omega$ being the complex-valued amplitude of the plane-wave electromagnetic vector potential of frequency $\omega \approx \Omega$, evaluated at the center of mass of the Cu(II) ion, and $\bar{n}$ being the effective refractive index of the sample. The local depolarization changes the local electric field incident on each Cu(II) ion to $\mathbf{E}_\omega(\bar{n}^2 + 2)/3$. Under the action of W, with $\mathbf{k}$ along $\mathbf{B}_0$, the expressions for P_{M1M1} and $P_{E1M1}^{D/L}$ read, re-

spectively, at leading order in the coupling constants of the interaction potentials,

$$P_{M1M1}|_{\omega\approx\Omega}=\hbar^{-2}\left|\int_0^{\mathcal{T}}\mathrm{d}te^{-i(\mathcal{T}-t)(\Omega/2-i\Gamma/2)}e^{-it(\omega-\Omega/2)}\langle\Phi|-g\mu_B\mathbf{S}\cdot\mathbf{B}_\omega|\Psi\rangle\right|^2$$
$$-\hbar^{-2}\left|\int_0^{\mathcal{T}}\mathrm{d}te^{-i(\mathcal{T}-t)(2\omega-\Omega/2-i\Gamma/2)}e^{-it(\omega+\Omega/2)}\langle\Phi'|-g\mu_B\mathbf{S}\cdot\mathbf{B}_\omega|\Psi\rangle\right|^2, \quad (7)$$

$$P^{D/L}_{E1M1}|_{\omega\approx\Omega}=2\mathrm{Re}(-i)^3\hbar^{-4}\sum_{p,q\neq\Psi}\int_0^{\mathcal{T}}\mathrm{d}te^{-i(\mathcal{T}-t)(\Omega/2-i\Gamma/2)}\langle\Phi|-e\mathbf{r}\cdot(\bar{n}^2+2)\mathbf{E}_\omega/3|p\rangle\int_{-\infty}^{t}\mathrm{d}t'e^{\eta t'}e^{-i(t-t')(\mathcal{E}_p+\omega)}$$
$$\times\langle p|V_C^{D/L}|q\rangle\int_{-\infty}^{t'}\mathrm{d}t''e^{\eta t''}e^{-i(t'-t'')(\mathcal{E}_q+\omega)}\langle q|V_{SO}|\Psi\rangle e^{-it''(\omega-\Omega/2)}i\int_0^{\mathcal{T}}\mathrm{d}\tau\, e^{i(\mathcal{T}-\tau)(\Omega/2+i\Gamma/2)}$$
$$\times\langle\Psi|-g\mu_B\mathbf{S}\cdot\mathbf{B}^*_\omega|\Phi\rangle e^{i\tau(\omega-\Omega/2)}+2\mathrm{Re}(-i)^3\hbar^{-4}\sum_{p,q\neq\Phi}\int_{-\infty}^{\mathcal{T}}\mathrm{d}t\, e^{\eta t}e^{-i(\mathcal{T}-t)(\Omega/2-i\Gamma/2)}\langle\Phi|V_{SO}|p\rangle$$
$$\times\int_{-\infty}^{t}\mathrm{d}t'e^{\eta t'}e^{-i(t-t')\mathcal{E}_p}\langle p|V_C^{D/L}|q\rangle\int_0^{t'}\mathrm{d}t''e^{-i(t'-t'')\mathcal{E}_q}\langle q|-e\mathbf{r}\cdot(\bar{n}^2+2)\mathbf{E}_\omega/3|\Psi\rangle e^{-it''(\omega-\Omega/2)}$$
$$\times i\int_0^{\mathcal{T}}\mathrm{d}\tau\, e^{i(\mathcal{T}-\tau)(\Omega/2+i\Gamma/2)}\langle\Psi|-g\mu_B\mathbf{S}\cdot\mathbf{B}^*_\omega|\Phi\rangle e^{i\tau(\omega-\Omega/2)}$$
$$-2\mathrm{Re}(-i)^3\hbar^{-4}\sum_{p,q\neq\Psi}\int_0^{\mathcal{T}}\mathrm{d}t\, e^{-i(\mathcal{T}-t)(2\omega-\Omega/2)}\langle\Phi'|-e\mathbf{r}\cdot(\bar{n}^2+2)\mathbf{E}_\omega/3|p\rangle\int_{-\infty}^{t}\mathrm{d}t'e^{\eta t'}e^{-i(t-t')(\mathcal{E}_p+\omega)}$$
$$\times\langle p|V_C^{D/L}|q\rangle\int_{-\infty}^{t'}\mathrm{d}t''e^{\eta t''}e^{-i(t'-t'')(\mathcal{E}_q+\omega)}\langle q|V_{SO}|\Psi\rangle e^{-it''(\omega+\Omega/2-i\Gamma/2)}i\int_0^{\mathcal{T}}\mathrm{d}\tau\, e^{i(\mathcal{T}-\tau)(2\omega-\Omega/2)}$$
$$\times\langle\Psi|-g\mu_B\mathbf{S}\cdot\mathbf{B}^*_\omega|\Phi'\rangle e^{i\tau(\omega+\Omega/2+i\Gamma/2)}-2\mathrm{Re}(-i)^3\hbar^{-4}\sum_{p,q\neq\Phi'}\int_{-\infty}^{\mathcal{T}}\mathrm{d}t\, e^{\eta t}e^{-i(\mathcal{T}-t)(2\omega-\Omega/2)}$$
$$\times\langle\Phi'|V_{SO}|p\rangle\int_{-\infty}^{t}\mathrm{d}t'e^{\eta t'}e^{-i(t-t')(2\omega+\mathcal{E}_p)}\langle p|V_C^{D/L}|q\rangle\int_0^{t'}\mathrm{d}t''e^{-i(t'-t'')(2\omega+\mathcal{E}_q)}$$
$$\times\langle q|-e\mathbf{r}\cdot(\bar{n}^2+2)\mathbf{E}_\omega/3|\Psi\rangle e^{-it''(\omega+\Omega/2-i\Gamma/2)}i\int_0^{\mathcal{T}}\mathrm{d}\tau\, e^{i(\mathcal{T}-\tau)(2\omega-\Omega/2)}\langle\Psi|-g\mu_B\mathbf{S}\cdot\mathbf{B}^*_\omega|\Phi'\rangle$$
$$\times e^{i\tau(\omega+\Omega/2+i\Gamma/2)},\quad \eta\rightarrow0^+,\ \Gamma\mathcal{T}\gg1. \quad (8)$$

In these equations the states p and q stand for the excited states of the $3d^9$ configuration together with other eigenstates of H_0 with $n\neq2$. The quasi-stationary condition $\eta\rightarrow0^+$ accounts for the stationarity of the chiral and the spin-orbit interactions; whereas the adiabatic limit $\Gamma\mathcal{T}\gg1$ takes into account the long duration of the probe field with respect to the lifetime Γ^{-1}, with Γ being the linewidth of absorption and $\mathcal{T}$ the observation time. The diagrammatical representation of the processes involved in the above equation is given in Fig.1. In the Letter, the contributions of the quasi-stationary processes were incorporated into the dressed states $\tilde{\Psi}$, $\tilde{\Phi}$, $\tilde{\Phi}'$. More specifically, the bare states are dressed with the

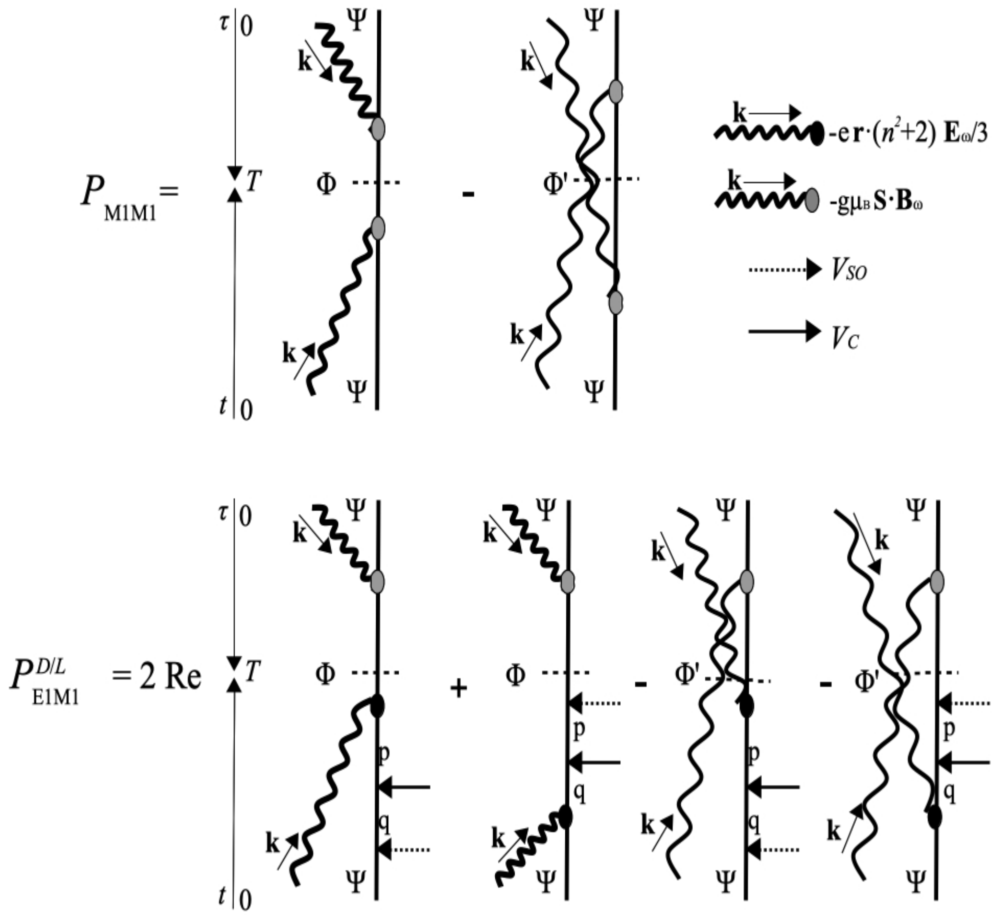

FIG. 1: Diagrammatic representation of the processes which contribute to P_{M1M1} and $P^{D/L}_{E1M1}$ for $\omega \approx \Omega$ at leading order in the perturbative interactions, i.e., at second order and fourth order, respectively. Time runs along the vertical direction from 0 to the observation time $\mathcal{T}$, where the probability is computed. Intermediate atomic states are labeled as p and q. Diagrams with two-photon states account for stimulated emission.

quadruplet $\{\Phi_1, .., \Phi_4\}$ through V_{SO}, and with harmonic states with $n \neq 2$ by V_C. In terms

of the eigenstates of the harmonic oscillator, they read

$$
\begin{aligned}
|\tilde{\Phi}\rangle =& \Big[(|020\rangle + |200\rangle)/\sqrt{2} + \frac{\lambda}{\sqrt{2}\Delta_0}(1+\Delta_2/\Delta_0)(|020\rangle - |200\rangle) + \frac{i\lambda C^{D/L}K^{3/2}}{2\hbar\omega_0\Delta_0}(1+\Delta_2/\Delta_0) \\
&\times (|001\rangle - 2|111\rangle)\Big]\downarrow + \Big[\frac{\lambda}{\sqrt{2}\Delta_0}(1+3\hbar\Omega/\Delta_0)|011\rangle + \frac{\delta}{\sqrt{2}\Delta_0^2}(\hbar\Omega+\lambda/2)|101\rangle \\
&+ \frac{-iC^{D/L}K^{3/2}\lambda}{2\hbar\omega_0\Delta_0}(1+3\hbar\Omega/\Delta_0)(|210\rangle - \sqrt{3}|030\rangle - \sqrt{2}|100\rangle) + \frac{iC^{D/L}K^{3/2}\delta}{2\hbar\omega_0\Delta_0^2} \\
&\times (\hbar\Omega+\lambda/2)(|120\rangle - \sqrt{3}|300\rangle - \sqrt{2}|010\rangle)\Big]\uparrow \\
|\tilde{\Phi}'\rangle =& \Big[(|020\rangle + |200\rangle)/\sqrt{2} + \frac{-\lambda}{\sqrt{2}\Delta_0}(1+\Delta_2/\Delta_0)(|020\rangle - |200\rangle) + \frac{-i\lambda C^{D/L}K^{3/2}}{2\hbar\omega_0\Delta_0}(1+\Delta_2/\Delta_0) \\
&\times (|001\rangle - 2|111\rangle)\Big]\uparrow + \Big[\frac{-\lambda}{\sqrt{2}\Delta_0}(1-3\hbar\Omega/\Delta_0)|101\rangle + \frac{\delta}{\sqrt{2}\Delta_0^2}(\hbar\Omega-\lambda/2)|011\rangle \\
&+ \frac{-iC^{D/L}K^{3/2}\lambda}{2\hbar\omega_0\Delta_0}(1-3\hbar\Omega/\Delta_0)(|120\rangle - \sqrt{3}|300\rangle - \sqrt{2}|010\rangle) + \frac{-iC^{D/L}K^{3/2}\delta}{2\hbar\omega_0\Delta_0^2} \\
&\times (\hbar\Omega-\lambda/2)(|210\rangle - \sqrt{3}|030\rangle - \sqrt{2}|100\rangle)\Big]\downarrow \\
|\tilde{\Psi}\rangle =& \cos\theta/2|\tilde{\Phi}'\rangle + \sin\theta/2|\tilde{\Phi}\rangle, \qquad K = \hbar/(2m_e\omega_0). \qquad (9)
\end{aligned}
$$

Using a linearly polarized incident field and averaging in orientations around the $\hat{\mathbf{z}}$-axis, we obtain, for $\lambda \gg \delta$,

$$P_{M1M1}|_{\omega\approx\Omega} \simeq \frac{\hbar^{-2}\mu_B^2|B_\omega|^2}{4[(\omega-\Omega)^2+\Gamma^2/4]}\cos\theta, \qquad (10)$$

$$P_{E1M1}^{D/L}|_{\omega\approx\Omega} \simeq \frac{(\bar{n}^2+2)}{3}\frac{C^{D/L}\Omega\delta}{m_e\omega_0^3\Delta_0^2}\frac{\hbar^{-1}\mu_B^2|B_\omega||E_\omega|}{4[(\omega-\Omega)^2+\Gamma^2/4]}\cos\theta, \qquad (11)$$

$$g_T^{D/L} \simeq \frac{(\bar{n}^2+2)}{3\bar{n}}\frac{c\,C^{D/L}\hbar\Omega\delta}{m_e\omega_0^3\Delta_0^2} + \mathcal{O}(\delta/\lambda, \lambda/\Delta_0). \qquad (12)$$

Lastly, it is worth mentioning that for the case $\delta > \lambda$, i.e., when anisotropy dominates over the spin-orbit coupling, $g_T^{D/L}$ scales as $(c\hbar C^{D/L}\Omega\delta\lambda)/(m_e\omega_0^3\Delta_0^3)$ instead. This scenario will be addressed in a separate publication [7].

Note 2b. Optical MChD

Optical MChD involves transitions of frequency Δ_0 from the ground state $|\Psi\rangle$ to the quasi-degenerate quadruplet $\{|d_{zx}\rangle, |d_{yz}\rangle\} \otimes \{\uparrow, \downarrow\}$ which, in account of the Zeeman and spin-orbit interactions, for $\delta \ll \lambda$, corresponds to the set of states $\{\Phi_1, ..., \Phi_4\}$ of Eq.(2). In contrast to EPR, the absorption probability in the denominator of the ratio $g_O^{D/L} = [P^{D/L}(\omega, \hat{\mathbf{k}}, \mathbf{B}_0) - P^{D/L}(\omega, \hat{\mathbf{k}}, -\mathbf{B}_0)]/[P^{D/L}(\omega, \hat{\mathbf{k}}, \mathbf{B}_0) + P^{D/L}(\omega, \hat{\mathbf{k}}, -\mathbf{B}_0)]$ for $\omega \approx \Delta_0/\hbar$ may

not be dominated by the magnetic-magnetic dipole absorption probability. This might be so because the d-orbitals of the Cu(II) ion hybridize generally with the σ and π orbitals of the ligands, allowing for additional electric-electric dipole (E1E1) transitions. For the sake of simplicity, we will neglect the latter in our calculations, which implies that our preliminar estimate for $g_O^{D/L}$ must be intended as an approximate upper bound. As for the case of EPR, the numerator of the ratio in $g_O^{D/L}$ is again dominated by the electric-magnetic dipole absorption probability, and the non-vanishing terms come from magnetic transitions driven by the spin angular momentum –Eq.(14) below. However, in contrast to EPR, the magnetic transitions in the denominator are mainly driven by the orbital angular momentum operator –see Eq.(13) below. In turn, this causes the E1M1 transition probability to depend on the spin polarization of the complex, whereas neither the M1M1 nor the E1E1 probabilities do. Note also that stimulated emission from the state $|\Psi\rangle$ is absent in optical MChD. All in all, this implies that $g_O^{D/L}$ is proportional to the magnetization of the sample, which is itself proportional to the degree of spin-polarization along $\mathbf{B}_0$, $\cos\theta$, in agreement with experiments. In Fig.2 we depict the diagrams which contribute at leading order to P_{M1M1} and $P_{E1M1}^{D/L}$ in optical MChD. Following a perturbative approach analogous to that in EPR [12], for an incident electromagnetic plane wave with $\mathbf{k} \parallel \mathbf{B}_0$ and assuming $\delta \ll \lambda$, one arrives at

$$P_{M1M1}|_{\omega\approx\Delta_0/\hbar} \simeq \frac{\hbar^{-2}\mu_B^2|B_\omega|^2}{4[(\omega-\Delta_0/\hbar)^2+\Gamma'^2/4]}, \tag{13}$$

$$P_{E1M1}^{D/L}|_{\omega\approx\Delta_0/\hbar} \simeq \frac{(\bar{n}^2+2)}{3}\frac{C^{D/L}\delta}{2m_e\omega_0^3\tilde{\Delta}}\frac{\hbar^{-2}\mu_B^2|B_\omega||E_\omega|}{4[(\omega-\Delta_0/\hbar)^2+\Gamma'^2/4]}\cos\theta, \tag{14}$$

$$g_O^{D/L} \lesssim \frac{P_{E1M1}^{D/L,O}}{P_{M1M1}^{O}}\Big|_{\omega\approx\Delta_0/\hbar} \simeq \frac{(\bar{n}^2+2)}{3\bar{n}}\frac{c\,C^{D/L}\delta\cos\theta}{2\,m_e\omega_0^3\tilde{\Delta}}, \tag{15}$$

where $\tilde{\Delta}^{-1} = \Delta_0^{-1} + \Delta_2^{-1} - 3\Delta_1^{-1}$, and Γ' is the linewidth of optical absorption. As anticipated, the fact that the magnetic dipole transition in $P_{E1M1}^{D/L}$ is dominated by the orbital angular momentum operator causes its leading order term to depend on the magnetization $\sim \cos\theta$. Hence, time-reversal invariance happens to be broken by the spin-polarization of the complex.

Note 2c. Estimate of $g_T^{D/L}$

In the first place, we work out the relationship between $g_T^{D/L}$ and $g_O^{D/L}$. Comparing Eq.(11) with Eq.(14) at resonance, and taking into account Eqs.(12) and (15), we arrive at the

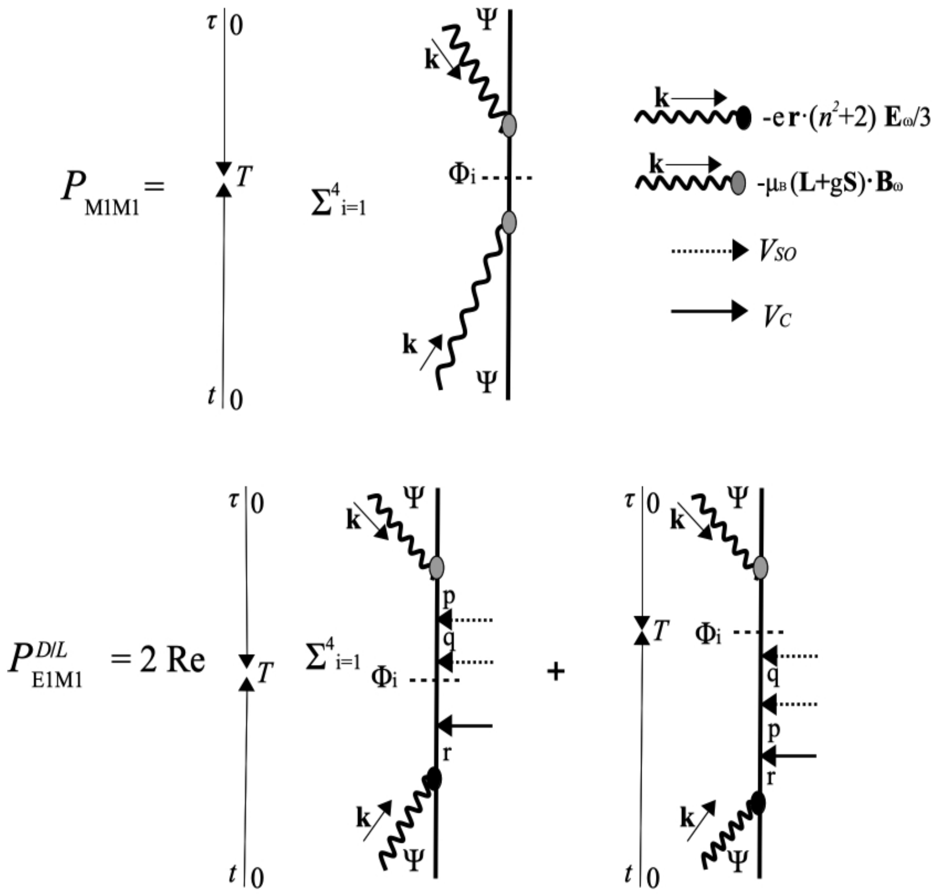

FIG. 2: Diagrammatic representation of P_{M1M1} and $P_{E1M1}^{D/L}$ for $\omega \approx \Delta_0/\hbar$ at leading order in the perturbative interactions, i.e., at second and up to fifth order, respectively. Intermediate atomic states are labeled as p, q, r, s.

following relationships,

$$\frac{P_{E1M1}^{D/L}|_{\omega=\Omega}}{P_{E1M1}^{D/L}|_{\omega=\Delta_0/\hbar}} \simeq \frac{2\hbar\Omega\tilde{\Delta}\Gamma'^2}{\Delta_0^2\Gamma^2}, \qquad \frac{g_T^{D/L}}{g_O^{D/L}} \gtrsim \frac{2\hbar\Omega\tilde{\Delta}}{\Delta_0^2\cos\theta}. \tag{16}$$

Next, considering the experimental data obtained in Ref.[4] for $g_O^{D/L}$ and applying the relationship in Eq.(16), we can estimate a lower bound for $g_T^{D/L}$. That is, substituting into Eq.(16) the experimental values $g_O^{D/L} \approx 0.025$, $\cos\theta \approx 0.4$, for $B_0 = 14$T at a temperature of 4.2 K, we obtain $g_T^{D/L} \gtrsim 10^{-4}$.

Alternatively, we can estimate $g_T^{D/L}$ using the experimental data of Ref.[4] for the non-reciprocal absorption coefficient of optical MChD, $\alpha_A = \alpha(\mathbf{B}_0 \upharpoonleft\upharpoonright \mathbf{k}) - \alpha(\mathbf{B}_0 \downharpoonleft\upharpoonright \mathbf{k})$. In order

to do so, we first write down α_A as a function of $P^{D/L,O}_{E1M1}$ at resonance,

$$\alpha_A = \frac{4c\mu_0\rho\Gamma'\Delta_0}{|E_\omega|^2} P^{D/L}_{E1M1}|_{\omega=\Delta_0/\hbar}, \tag{17}$$

where ρ is the molecular density of the $CsCuCl_3$ complex (mass density $3.5 g/cm^3$). Substituting the expression for $P^{D/L,O}_{E1M1}(\omega = \Delta_0/\hbar)$ in the above equation and using Eq.(12) we arrive at the equalities,

$$C^{D/L}\delta = \frac{3\hbar^2 m_e \omega_0^3 \tilde{\Delta}\Gamma'\alpha_A}{2(\bar{n}^2+2)\rho\mu_0\mu_B^2\Delta_0\cos\theta}, \quad g_T^{D/L} = \frac{c\,\hbar^3\Gamma'\Omega\tilde{\Delta}\alpha_A}{2\Delta_0^3\mu_0\mu_B^2\rho\cos\theta}. \tag{18}$$

Substituting the experimental values for all the variables in Eq.(18), for $B_0 = 14\,\mathrm{T}$ at a temperature of $4.2\,\mathrm{K}$, with $\Gamma' \approx 0.1\mathrm{eV}$ and $\bar{n} \approx 1.5$, we obtain $g_T^{D/L} \approx 1.5 \cdot 10^{-2}$, in agreement with our previous lower bound estimate.

Note 3. Further comments on the Hamiltonian model

Despite the success of our model to provide analytical estimates for the MChA factors, there is still room for improvement. In the first place, concerning the chiral Hamiltonian V_C, it was written in terms of the local axis of the octahedral structure, x, y, z, while it should be adapted to the crystal axes to account for the helical distribution of the active ions along the c-axis. In fact, the experimental data on α_A taken from the literature to estimate $g_T^{D/L}$ consider $\mathbf{B}_0$ along the c-axis. Also, the harmonic oscillator model, which is considered only distorted in the $n = 2, l = 2$ level, may not be accurate enough to account for the intermediate transitions induced by the chiral potential to levels with $n \neq 2$. Hence, a more accurate confining potential model, though less generic, can be obtained using a more detailed formulation of the crystal field and the JT distortion for the particular case of $CsCuCl_3$–see, eg., Ref.[10]. Also, in our calculations we constrained ourselves to the single-molecule approximation, meaning that we neglected electrostatic and magnetostatic interactions between nearby molecules. The inclusion of the former would imply additional near-field factors in the local field factors which accompany the formulas that depend on the electric field [e.g., Eqs.(12), (14), (17)]. For $\bar{n} \approx 1.5$, when evaluated at the absorption frequency, those factors must depend on the density ρ and the spatial correlations between molecules [11]. The incorporation of the magnetostatic interaction would imply the addition of terms in the Hamiltonian which account for the a spin-spin coupling between neighbouring

Cu^{+2} ions.

[1] Condon, E. U. Theories of optical rotatory power. *Rev. Mod. Phys.* **1937**, *9*, 432-457.

[2] Condon, E. U.; Altar, W; Eyring, H. One-electron rotatory power. *J. Chem. Phys.* **1937**, *5*, 753-775.

[3] Donaire, M.; Rikken, G. L.J.A.; van Tiggelen, B. A. A single-oscillator quantum model for magnetochiral birefringence. *Eur. Phys. J. D* **2014**, *68*, 33.

[4] Nakagawa, N.; *et al.* Magneto-chiral dichroism of $CsCuCl_3$. *Phys. Rev. B* **2017**, *96*, 121102(R).

[5] Tanaka, H.; Schotte, U.; Schotte, K.D. ESR modes in $CsCuCl_3$. *J. Phys. Soc. Japan* **1992**, *61*, 13441350.

[6] Cohen-Tannoudji, C.; Diu, B.; Laloe, F. Quantum mechanics. Wiley-VCH, New York ,1992.

[7] Donaire, M.; Rikken, G. L.J.A. In preparation.

[8] Toyoda, S.; Abe, N.; Kimura, S.; Matsuda, Y. H.; Nomura, T.; Ikeda, A.; Takeyama, S.; Arima, T. One-way transparency of light in multiferroic CuB_2O_4. *Phys. Rev. Lett.* **2015**, *115*, 267207; Sera, A.; Kousaka, Y.; Akimitsu, J.; Sera, M.; Kawamata, T.; Koike, Y.; Inoue, K. S=12 triangular-lattice antiferromagnets $Ba_3CoSb_2O_9$ and $CsCuCl_3$: Role of spin-orbit coupling, crystalline electric field effect, and Dzyaloshinskii-Moriya interaction. *Phys. Rev. B* **2016**, *94*, 214408.

[9] Ariponnammal, S.; Velvizhi, R. Structural, spectroscopic, and magnetic studies on copper tartrate crystals. *Z. Naturforsch*, **2019**, *74*, 813-819.

[10] Maaskant, W. J. A.; Haije, W. G. J. On the Jahn-Teller-induced helical deformations in $CsCuCl_3$. *Phys. C: Solid State Phys.*, **1986**, *19*, 5295-5308.

[11] Donaire, M. Electromagnetic vacuum of complex media: Dipole emission versus light propagation, vacuum energy, and local field factors. *Phys. Rev. A* **2011**, *83*, 022502.

[12] Note that, in contrast to the analogous processes in EPR, the spin-orbit potential enters at order 2. The reason being that the leading SO corrections to the states within the multiplet $\{\Phi_1, .., \Phi_4\}$ involve SO transitions within the multiplet. Since their energy differences are already of order λ, the first order SO corrections comprise up to V_{SO}^2 terms.
